# PreMeta: a tool to facilitate meta-analysis of rare-variant associations

**DOI:** 10.1186/s12864-017-3573-1

**Published:** 2017-02-14

**Authors:** Zheng-Zheng Tang, Paul Bunn, Ran Tao, Zhouwen Liu, Dan-Yu Lin

**Affiliations:** 1 Department of Biostatistics, Vanderbilt University School of Medicine, Nashville, 37203 TN USA; 2 Department of Biostatistics, University of North Carolina, Chapel Hill, 37203 NC USA

**Keywords:** MASS, MetaSKAT, RAREMETAL, seqMeta, Summary statistics, Gene-based association tests

## Abstract

**Background:**

Meta-analysis is essential to the discovery of rare variants that influence complex diseases and traits. Four major software packages, namely MASS, MetaSKAT, RAREMETAL, and seqMeta, have been developed to perform meta-analysis of rare-variant associations. These packages first generate summary statistics for each study and then perform the meta-analysis by combining the summary statistics. Because of incompatible file formats and non-equivalent summary statistics, the output files from the study-level analysis of one package cannot be directly used to perform meta-analysis in another package.

**Results:**

We developed a computationally efficient software program, PreMeta, to resolve the non-compatibility of the four software packages and to facilitate meta-analysis of large-scale sequencing studies in a consortium setting. PreMeta reformats the output files of study-level summary statistics generated by the four packages (text files produced by MASS and RAREMETAL, binary files produced by MetaSKAT, and R data files produced by seqMeta) and translates the summary statistics from one form to another, such that the summary statistics from any package can be used to perform meta-analysis in any other package. With this tool, consortium members are not required to use the same software for study-level analyses. In addition, PreMeta checks for allele mismatches, corrects summary statistics, and allows the rescaled inverse normal transformation to be performed at the meta-analysis stage by rescaling summary statistics.

**Conclusions:**

PreMeta processes summary statistics from the four packages to make them compatible and avoids the need to redo study-level analyses. PreMeta documentation and executable are available at: http://dlin.web.unc.edu/software/premeta.

## Background

There is a growing interest in performing meta-analysis of sequencing studies to discover rare variants that influence complex diseases and traits. Four major software packages — MASS [1, 2], MetaSKAT [3], RAREMETAL [4, 5], and seqMeta [6] — have been developed for this purpose. These packages perform two separate tasks: (1) *study-level analysis* — computation of summary statistics for each sequencing study; and (2) *meta-analysis* — combination of summary statistics to perform gene-based association tests. For MASS and RAREMETAL, the first step is supported by separate software programs SCORE-Seq/SCORE-SeqTDS [7, 8] and RAREMETALWORKER/RVTESTS, respectively. For MetaSKAT and seqMeta, the first step is supported by certain functions in the corresponding R packages. To simplify description, we will use the name MASS or RAREMETAL to denote both the study-level analysis programs and the meta-analysis software itself when the distinction is not necessary.

Because of incompatible file formats and non-equivalent summary statistics, the output files from the study-level analysis of one package cannot be directly used to perform meta-analysis in another package. Thus, all participating studies in a consortium are required to use the same package. This requirement is highly undesirable for several reasons. First, participating investigators may not be familiar with the package selected by the consortium. Second, no single package can handle all types of traits (e.g., binary and survival traits) or study designs (e.g., family studies and extreme-trait sampling). Third, summary statistics need to be recalculated when investigators join a new consortium that adopts a different package.

To avoid the aforementioned requirement, we developed a C++ program called PreMeta. This program converts between the file formats of the four packages and translates the summary statistics to a common form, so that the summary statistics from different packages can be properly combined for meta-analysis. PreMeta operates as an open source stand-alone software program designed for easy installation, simple interface, and high performance. With this tool, participating studies are allowed to use the packages of their choice, and the results can be used in future meta-analysis without re-doing the study-level analysis.

## Implementation

The files of summary statistics generated by the four software packages have different formats. Specifically, MASS uses one text file to provide score statistics, gene-based covariance matrices, and genotype information, including minor allele frequency (MAF), minor allele count, count of non-missing genotypes, and counts of homozygous reference, heterozygous, and homozygous alternative genotypes. RAREMETAL uses two compressed text files: the.COV file tabulates sliding-window covariances; and the.SCORE file contains score statistics, genotype information (i.e., reference and alternative alleles, Hardy-Weinberg Equilibrium (HWE) *p*-value, and the information in the MASS file), and trait information (i.e., mean, standard deviation, percentiles). MetaSKAT uses two specialized files:.MSSD is a binary file with gene-based covariance matrices; and.MInfo is a text file with score statistics and genotype information (i.e., reference and alternative alleles, MAF, missing rate). seqMeta uses an R data file containing score statistics, gene-based covariance matrices, MAF, and standard error of the residuals. A major function of PreMeta is to convert between the four file formats.

The four software packages present the score statistics and covariance information in various forms. For each gene, MASS and MetaSKAT provide the score statistic normalized by the residual variance and the corresponding covariance matrix. SeqMeta provides the unnormalized score statistics, their covariance matrix, and the residual variance. RAREMETAL provides the normalized score statistics and the corresponding covariances divided by the sample size in a sliding-window format. For studies of unrelated subjects, the older versions of RAREMETAL (e.g., v0.4.0) provides unnormalized score statistics. PreMeta translates the score statistics and covariance information from one form to another.

When converting the gene-based covariances to the sliding-window format, there is not enough information to produce the covariance between two single nucleotide polymorphisms (SNPs) that lie in one window but on two different genes. PreMeta sets the covariance to zero for any such SNP pair. This implementation will correctly recover the gene-based covariance in the meta-analysis if the same gene grouping is adopted.

Inverse normal transformation (INT) is commonly used to yield normal distributions for quantitative traits. This transformation alters the scale of measurement for the genetic effects and thus tends to reduce the power of meta-analysis. One may restore the original scale of measurement by multiplying the transformed trait values with the standard deviation of the original trait values. PreMeta allows this rescaled INT (R-INT) to be performed at the meta-analysis stage by rescaling the score statistics and covariance matrices.

The four packages generate a variety of auxiliary information, which may not be equivalent. If a particular field in the target files is essential for the corresponding software to perform meta-analysis but cannot be retrieved or derived from the provided files, then PreMeta will request additional information from the user. For instance, the reference and alternative alleles are required for meta-analysis by RAREMETAL and MetaSKAT but are not available in the files of MASS and seqMeta. In this case, PreMeta will request an additional file with the allele information when converting files from the MASS or seqMeta format to the RAREMETAL or MetaSKAT format. If a particular field in the target files is not essential for meta-analysis, then PreMeta will recover this field whenever possible and insert pseudo values that are compatible with the target format otherwise. For example, the HWE *p*-value is provided by RAREMETAL but not by the other three packages. PreMeta will calculate the HWE *p*-value based on the heterozygous and homozygous counts when converting files from the MASS format and will set it to be one when converting files from the seqMeta or MetaSKAT format because the information in these files are not sufficient to calculate HWE *p*-value and RAREMETAL does not allow this value to be missing.

PreMeta is a freely available C++ program that runs on Unix and Linux systems. It is a stand-alone executable and can process (i.e., read/write) files produced by other packages: text files produced by MASS and RAREMETAL, binary files produced by MetaSKAT, and R data files produced by seqMeta. The basic command is:



The software argument specifies the target software, the version of which can be specified by the version argument. PreMeta currently supports four target software packages: v7.0 of MASS, v4.13.5 of RAREMETAL, v0.40 of MetaSKAT, and v1.5 of seqMeta. The script argument specifies a file that lists the input files to be converted by PreMeta and is formatted as described below.

**Fig. 1:**
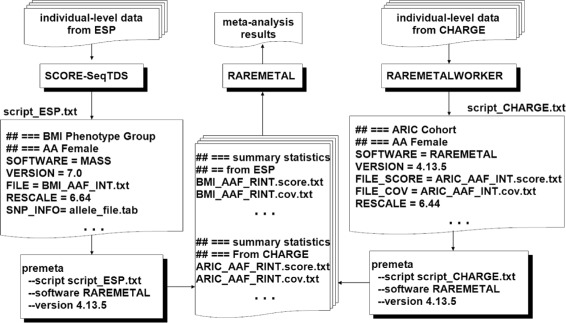
The analysis pipeline for the meta-analysis of all the ESP and CHARGE studies on body mass index, with the ESP phenotype groups and CHARGE cohorts stratified by race (AA: African American; EA: European American) and gender

The script file contains a block of keywords and values, with one block for each study; see Table 1 and Fig. 1. The keyword SOFTWARE indicates the software program that was used to generate the files for the study. This keyword is required to appear before all other keywords in each block. The keyword VERSION indicates the version of the software. PreMeta currently supports five software programs: v7.0 of MASS, v4.13.5 and v0.4.0 of RAREMETAL, v0.40 of MetaSKAT, and v1.5 of seqMeta. The old version of RAREMETAL (v0.4.0) is supported by PreMeta as input format to facilitate meta-analysis directly using the summary statistics in the old format of RAREMETAL and thus avoid re-running the study-level analysis to create files in the current format. The RAREMETAL v0.4.0 is not supported by PreMeta as output format because the current meta-analysis should use the new version of RAREMETAL. The files to be converted are specified with keywords prefixed by FILE ∗; the specific keywords depend on the software. If SOFTWARE = MASS, then the file from MASS should be specified via the keyword FILE. If SOFTWARE = RAREMETAL, then three files must be provided: the covariance and score files from RAREMETAL should be specified via the keywords FILE_COV and FILE_SCORE, respectively, and a gene grouping file must be specified via the keyword FILE_GROUP. This third file is necessary to convert the sliding-window covariances to the gene-based covariance matrices of the other three packages. The FILE_GROUP file is not necessary if the target software is also RAREMETAL, e.g., when using PreMeta’s RESCALE functionality or converting the old RAREMETAL format to the newer format. If SOFTWARE = MetaSKAT, then the.MSSD and.MInfo files should be specified via the keywords FILE_MSSD and FILE_MINFO, respectively. If SOFTWARE = seqMeta, then the RData containing the seqMeta object should be specified via the keyword FILE_RDATA. Finally, the keyword RESCALE is used to implement R-INT by specifying the original trait variance.

**Table 1:** Keywords and values in PreMeta script file

Keyword	Value
SOFTWARE	Software program for study-level analysis
VERSION	Version of the software
FILE	Name of the file generated by MASS
FILE_SCORE	Name of the score file generated by RAREMETAL
FILE_COV	Name of the covariance file generated by RAREMETAL
FILE_GROUP	Name of the file that defines the gene-level grouping
FILE_MSSD	Name of the MSSD file generated by MetaSKAT
FILE_MINFO	Name of the MINFO file generated by MetaSKAT
FILE_RDATA	Name of the RDATA file containing the SeqMeta object
RESCALE	Original trait variance to rescale summary statistics for INT
SNP_INFO	Name of the file listing reference and alternative alleles for all SNPs

An additional keyword SNP_INFO is required in the script file if the target software is RAREMETAL or MetaSKAT and the software for the study-level analysis is MASS or seqMeta. This file specifies the reference and alternative alleles for all SNP positions. An optional function of the SNP_INFO file is to provide mapping between the study-specific SNP ID and meta-analysis SNP ID, such that PreMeta can unify different forms of the SNP ID across studies. For example, the SNP ID in the RAREMETAL takes the form of chr:pos, so SNP_INFO needs to contain the meta-analysis SNP ID in the form of chr:pos if the study-specific SNP ID is not in this form.

PreMeta identifies allele mismatch across studies. It generates an allele reference list from the input files using the reference and alternative alleles of the first occurrence as the gold standard. Such list can also be provided by specifying the global_snp argument on the command line, in which case PreMeta flags the reference and alternative alleles that are not consistent with the list. In addition, PreMeta corrects the relevant summary statistics on the occasion that the reference and alternative alleles are switched.

## Results and discussion

PreMeta played an important role in the meta-analysis of exome sequencing data from the National Heart, Lung, and Blood Institute (NHLBI) Exome Sequencing Project (ESP) and the Cohorts for Heart and Aging Research in Genomic Epidemiology (CHARGE) consortium. RAREMETAL was chosen to perform meta-analysis of rare-variant associations. However, RAREMETAL is not appropriate for the NHLBI ESP because it does not support extreme-trait sampling. In the NHLBI ESP, 619, 601, and 692 individuals were sequenced because of extremely high or low values of body mass index, low-density lipoprotein, and blood pressure, respectively. Thus, we used SCORE-SeqTDS to generate summary statistics for the NHLBI ESP and RAREMETALWORKER for the CHARGE studies. We then used PreMeta to convert the files from the MASS format to the RAREMETAL format and rescaled the score statistics and covariances to implement R-INT for all studies. Finally, we uploaded the resulting files in RAREMETAL to perform meta-analysis. Figure 1 shows this analysis pipeline.

**Fig. 2:**
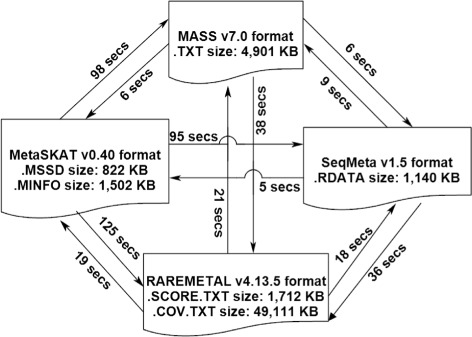
PreMeta runtime in reformatting summary statistics from the DPR female study

To evaluate the performance of PreMeta, we used the four packages to generate summary statistics for the female European Americans in the ESP’s deeply phenotyped reference (DPR), which is a random sample of individuals with measurements on a common set of phenotypes. We used PreMeta to convert the output files from one format to the other formats. Figure 2 shows the sizes of the output files from different packages when analyzing chromosome 1 (2105 genes, 191,134 variants). It also shows the PreMeta runtime for converting between any two formats on an IBM HS22 machine, which amounted to essentially a few seconds. The original prototype of PreMeta was written in an R script. The processing speed using the R script is slow in the real-data analysis, which prompted us to program PreMeta and its speed is much satisfactory.

**Fig. 3:**
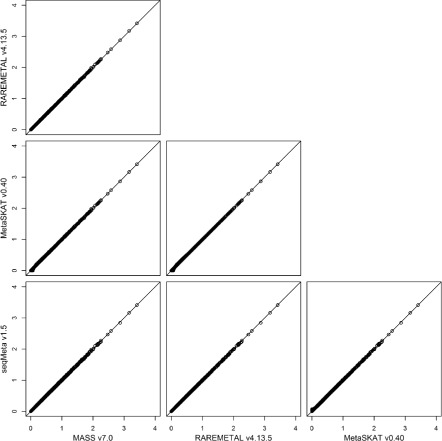
SKAT *p*-values in -log10 scale from meta-analysis of the DPR male and female studies by MASS v7.0, RAREMETAL v4.13.5, MetaSKAT v0.40, and seqMeta v1.5. The same software package was used to generate summary statistics for the studies of female and male European Americans in the DPR phenotype group of the NHLBI ESP and to perform the meta-analysis

**Fig. 4:**
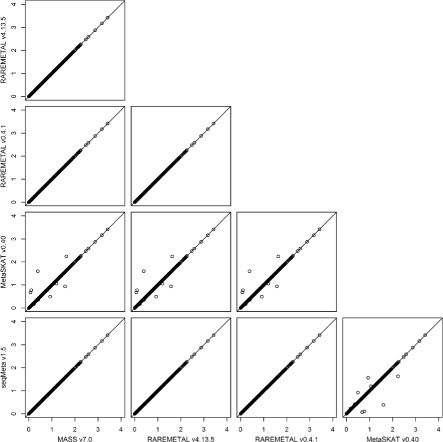
SKAT *p*-values in -log10 scale from meta-analysis of the DPR male and female studies by MASS v7.0. The summary statistics of the female study were generated in turn by MASS v7.0, RAREMETAL v4.13.5, RAREMETAL v0.4.0, MetaSKAT v0.40, and seqMeta v1.5. PreMeta converted these summary statistics to the MASS v7.0 format. MASS v7.0 was used to generate the summary statistics of the male study and to perform the meta-analysis by combining the reformatted summary statistics from each software package for the female study and the summary statistics for the male study

**Fig. 5:**
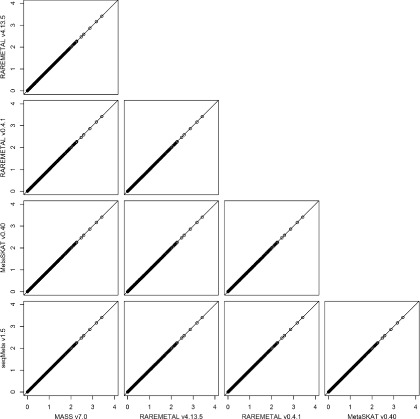
SKAT *p*-values in -log10 scale from meta-analysis of the DPR male and female studies by MetaSKAT v0.40. The summary statistics of the female study were generated in turn by MASS v7.0, RAREMETAL v4.13.5, RAREMETAL v0.4.0, MetaSKAT v0.40, and seqMeta v1.5. PreMeta converted these summary statistics to the MetaSKAT v0.40 format. MetaSKAT v0.40 was used to generate the summary statistics of the male study and to perform the meta-analysis by combining the reformatted summary statistics from each software package for the female study and the summary statistics for the male study

**Fig. 6:**
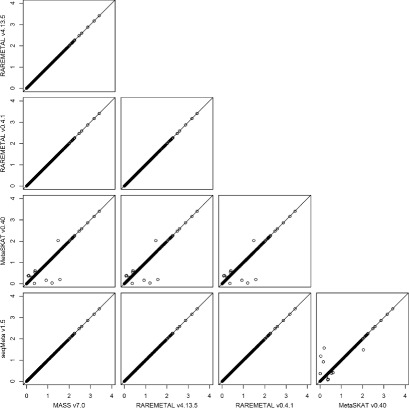
SKAT *p*-values in -log10 scale from meta-analysis of the DPR male and female studies by RAREMETAL v4.13.5. The summary statistics of the female study were generated in turn by MASS v7.0, RAREMETAL v4.13.5, RAREMETAL v0.4.0, MetaSKAT v0.40, and seqMeta v1.5. PreMeta converted these summary statistics to the RAREMETAL v4.13.5 format. RAREMETAL v4.13.5 was used to generate the summary statistics of the male study and to perform the meta-analysis by combining the reformatted summary statistics from each software package for the female study and the summary statistics for the male study

**Fig. 7:**
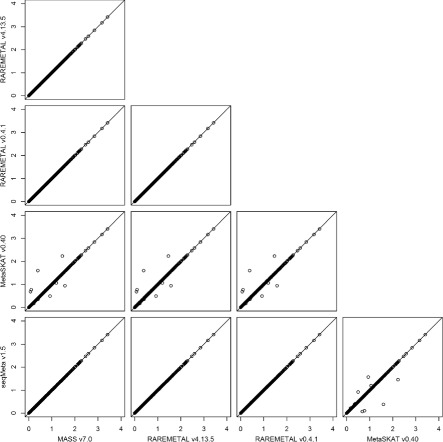
SKAT *p*-values in -log10 scale from meta-analysis of the DPR male and female studies by seqMeta v1.5. The summary statistics of the female study were generated in turn by MASS v7.0, RAREMETAL v4.13.5, RAREMETAL v0.4.0, MetaSKAT v0.40, and seqMeta v1.5. PreMeta converted these summary statistics to the seqMeta v1.5 format. seqMeta v1.5 was used to generate the summary statistics of the male study and to perform the meta-analysis by combining the reformatted summary statistics from each software package for the female study and the summary statistics for the male study

To investigate the intrinsic differences among the four packages and to illustrate the reformatting function of PreMeta, we performed meta-analysis of the female samples and the male samples from the DPR using the Sequence Kernel Association Test (SKAT). We first used each package to perform both the study-level analyses and the meta-analysis. The results from the four packages are almost identical (Fig. 3). We then converted the files for the female study from the formats of the other software packages to the format of the target package and repeated the analyses. We used the target package for the study-level analysis of the male study and the meta-analysis. The meta-analysis results based on the converted files are highly consistent with the results using files directly generated by the target package (Figs. 4, 5, 6 and 7). We examined the few inconsistent points, and they were all caused by the fact that MetaSKAT flipped reference and alternative alleles when MAF > 0.5 in the study-level analysis and removed SNPs with mismatched alleles among studies.

## Conclusions

Due to ethical and logistical difficulties in sharing individual-participant data, the use of summary statistics is strongly preferable when synthesizing results across studies. The summary statistics generated by the four software packages for meta-analysis of rare-variant associations are not compatible in format or content. PreMeta processes these summary statistics to make them compatible and avoids the need to redo study-level analyses. As different versions of a particular package may adopt different formats, we will periodically review the formats employed by new releases of the four packages and update PreMeta accordingly.

## Availability of data and materials

- **Project name:** PreMeta
- **Project home page:** http://dlin.web.unc.edu/software/premeta
- **Operating system(s):** Unix/Linux
- **Programming language:** C++
- **License:** GPL-3
- **Availability:** PreMeta, including source code, documentation, and examples, is freely available for non-commercial use with no restrictions at http://dlin.web.unc.edu/software/premeta.

## Abbreviations

CHARGE: Cohorts for heart and aging research in genomic epidemiology
DPR: Deeply phenotyped reference
ESP: Exome sequencing project
HWE: Hardy-weinberg equilibrium
INT: Inverse normal transformation
MAF: Minor allele frequency
NHLBI: National heart, lung, and blood institute
R-INT: Rescaled inverse normal transformation
SKAT: Sequence kernel association test
SNP: Single nucleotide polymorphism
